# Atypical HIV-vacuolar myelopathy: a case report

**DOI:** 10.1186/s40001-021-00483-0

**Published:** 2021-02-01

**Authors:** Tau Mongezi, Joseph Sibi, George Jerry, Ibañez-Valdés Lourdes de Fátima, Dubula Tozama, Foyaca Sibat Humberto

**Affiliations:** 1 Department of Internal Medicine, Nelson Mandela Academic Central Hospital (NMACH), Walter Sisulu University, Mthatha, South Africa; 2 Division of Neurology, NMACH, Walter Sisulu University, Mthatha, South Africa; 3 Department of Medicine, and Division of Internal Medicine, Water Sisulu University (WSU), Mthatha, South Africa

**Keywords:** Human immunodeficiency virus, Associated lesions of the nervous system, Human immunodeficiency virus-associated myelopathy, Intravenous immunoglobulin administration, Case report, HIV-vacuolar myelopathy

## Abstract

**Background:**

Here, we report an atypical HIV-vacuolar myelopathy and search the available medical literature about atypical presentations of human immunodeficiency virus associate vacuolar myelopathy (HIV-VM) and immunoglobulin therapy response.

**Case:**

A 26-year-old lady who was 4 weeks postpartum presented to us with acute flaccid quadriparesis, with no sensory level. Extensive workup ruled out other causes of myelopathy. She developed a stage 3 acute kidney injury, and MRI showed diffuse cord atrophy involving the lower cervical and thoracic cord. The patient received IV-immunoglobulin, ARVs, and supportive therapy with inadequate response. Unfortunately, she developed nosocomial pneumonia and died.

**Discussion:**

In HIV-VM, there is spinal cord atrophy, which mainly involves the thoracic cord. In our case, this pathological process also affected the spinal cord's cervical region, leading to flaccid tetraplegia, with high CD4 level, without response to the treatment, including intravenous immunoglobulin.

**Keynotes:**

Vacuolar myelopathy, HIV, Immunoglobulin therapy, flaccid tetraplegia, hypokalaemia. Renal failure.

## Background

HIV-associated vacuolar myelopathy (HIV-VM) is the most common and primary etiology of myelopathy in HIV/AIDS patients worldwide, leading to progressive spastic paralysis of the limbs, sensory ataxia, and autonomic dysfunction [1]. It derives its name from its pathological nature: formation of vacuoles mainly in the lateral and posterior columns of the spinal cord [2]. Some authors first reported this in 1985 [3]. Initially considered to present when HIV was in its advanced stages, as many authors had stated earlier on, even when the immunity was not suppressed [3]. The prevalence ranges from 22 to 55% [4], it does bear a poor prognosis [7], and due to its high pathological prevalence, it could also be underreported in the literature [5]. Up to date, the pathogenesis is not fully understood; it is essential to note that this is a diagnosis of exclusion requiring evaluation and elimination of other aetiologies [1–4]. Differential diagnoses include HIV-associated transverse myelitis during seroconversion, infections, e.g., viral—Herpes simplex (HSV), Varicella-Zoster (VZV), Cytomegalovirus (CMV), Human T-cell Lymphotropic Virus type 1 (HTLV-1/2); bacterial—Mycobacterium tuberculosis, neurosyphilis, multiple sclerosis, vitamin B12 deficiency, and compressive myelopathy, among others. MRI scans are useful in diagnosis; T2-weighted images often show symmetric non-enhancing high signal areas present on multiple contiguous slices, which result from extensive vacuolation (hence the name). Lesions may be confined to the posterior column, especially the gracile tracts, or may even be diffuse [3, 7]. Currently, there is no definitive treatment; however, most modalities focus on symptomatic therapies, combined antiretroviral treatment (cART) [9], and some authors have found some good outcomes prescribing IV-immunoglobulins [3, 10].

Here, we report a young female case in her postpartum stage who had an atypical HIV-VM presentation. She was a known HIV patient on cART, morbidly obese, confused, and quadriplegic with a history of renal failure and hypokalaemia that was corrected. Her viral load was suppressed and the CD4 + count was high. Unfortunately, she did not respond to IV-immunoglobulin therapy, which is relevant information for the medical community.

Our research questions were: how often IVIg is used to treat HIV-VM? How many positive results, including atypical presentations, have been published?

## Materials and methods

We searched for publications on HIV-vacuolar myelopathy and intravenous immunoglobulin therapy, in answer to the two research questions listed above using the procedure mentioned below and present our patient.

### Literature search strategy

For our literature review, we utilized the PRISMA (Preferred Reporting Items for Systemic review and Meta-Analysis) statement and the PRISMA checklist. We conducted the literature search from January 1, 2010, up to September 30, 2020. We included all studies (case reports, case series, and observational cohort studies) that reported HIV-VM and IVIG treatment during the initial search. We also reviewed the following databases for published studies: Medline EMBASE, Scopus online databases, Google Scholar, Science Direct, Scielo, LILACS, BIREME, and Cochrane library to identify articles evaluating HIV-VM and IVIg therapy*. All items about "AIDS-myelopathy* OR primary infectious myelopathy* OR HIV-VM* OR neurological manifestations of HIV/AIDS* OR Nosocomial myelopathy* OR Spinal cord syndrome/HIV/AIDS* OR Neuro-AIDS* OR Unknown cause myelopathy*OR infectious spinal cord disease* where * is the PubMed wildcard for every possible word beginning or ending. We did not consider other neurological manifestations beyond the scope of the current work.

### Study and cohort selection

We selected all publications (case reports, case series, and observational cohort studies) reporting HIV-VM, IVIg therapy during the initial search.

## Results

Between January 1, 2010, and September 30, 2020, our literature search yielded 621 publications. After removing duplicate articles, we retained 457 unique records. Considering the title and abstracts, we kept 38 items, thereafter screening full text. Finally, we found a total of 2 publications referring to HIV-VM and IVIg. We did not find any published study about poor response of HIV-VM after IVIg treatment from all groups.

## Case presentation

Ms. N Is a 26-year-old African lady admitted to Nelson Mandela Academic Central Hospital (NMACH) in Mthatha, South Africa. She presented in May 2020 with a history of inability to walk and confusion. We could not establish the duration of symptoms, signs, and the mode of onset from the admission chart, and she gave no further history due to her confused state.

Of note, this patient was still in her puerperal phase. She was admitted to the maternity ward at NMACH 2 months ago with severe preeclampsia and HELLP syndrome. This entity is an extreme form of preeclampsia characterized by hemolysis (H), elevated liver enzymes (EL), and low platelets (LP) in a pregnant or puerperal patient (usually within 7 days of delivery)]. Her clinical picture worsened by stage 3 acute kidney injury (creatinine was > 3 times the baseline), she had received intermittent hemodialysis, and renal function wholly recovered before discharge.

Her medical history shows, she was HIV positive, with a CD4:1051cell/µL and had a suppressed viral load (< 20 copies/ml) on a modified first-line regimen (ABC/3TC/EFV). She is also a known hypertensive patient on hydrochlorothiazide and amlodipine, and there is no previous history of target organ damage. However, according to her old chart, it was evident that she had defaulted on her antihypertensive treatment for about a month. Her family history was non-significant, and she was a non-smoker, never used illicit drugs, and occasionally used alcohol. She did not have any travel history outside Eastern Cape Province in South Africa.

On examination, we found her to be obese (BMI 36 kg/m^2^), with pink mucosal membranes, anicteric, and afebrile. Her vital signs demonstrated a tachycardia (heart rate: 130) and elevated BP: 147/109 mmHg. The patient was confused. No cranial nerve abnormalities or meningeal signs; she had bilateral mild horizontal vestibular nystagmus. Her motor examination revealed power of 0/5 in all limbs (proximally and distally), with hypotonia in all limbs and absent deep tendons reflexes. The sensory test was exceedingly difficult to perform due to her confusional state, but she seemed to respond to light touch and pain. On her respiratory examination, we confirmed fine crepitations in the right lower zones of the chest.

Given her acute presentation of flaccid quadriplegia, our differentials included Landry–Guillain–Barre syndrome and its variants, HIV-related neuropathies, metabolic derangements (hypokalaemia), vitamin B12 deficiency, CMV peripheral neuropathy, inflammatory myopathies, and neuromuscular junction disorders.

**Fig. 1:**
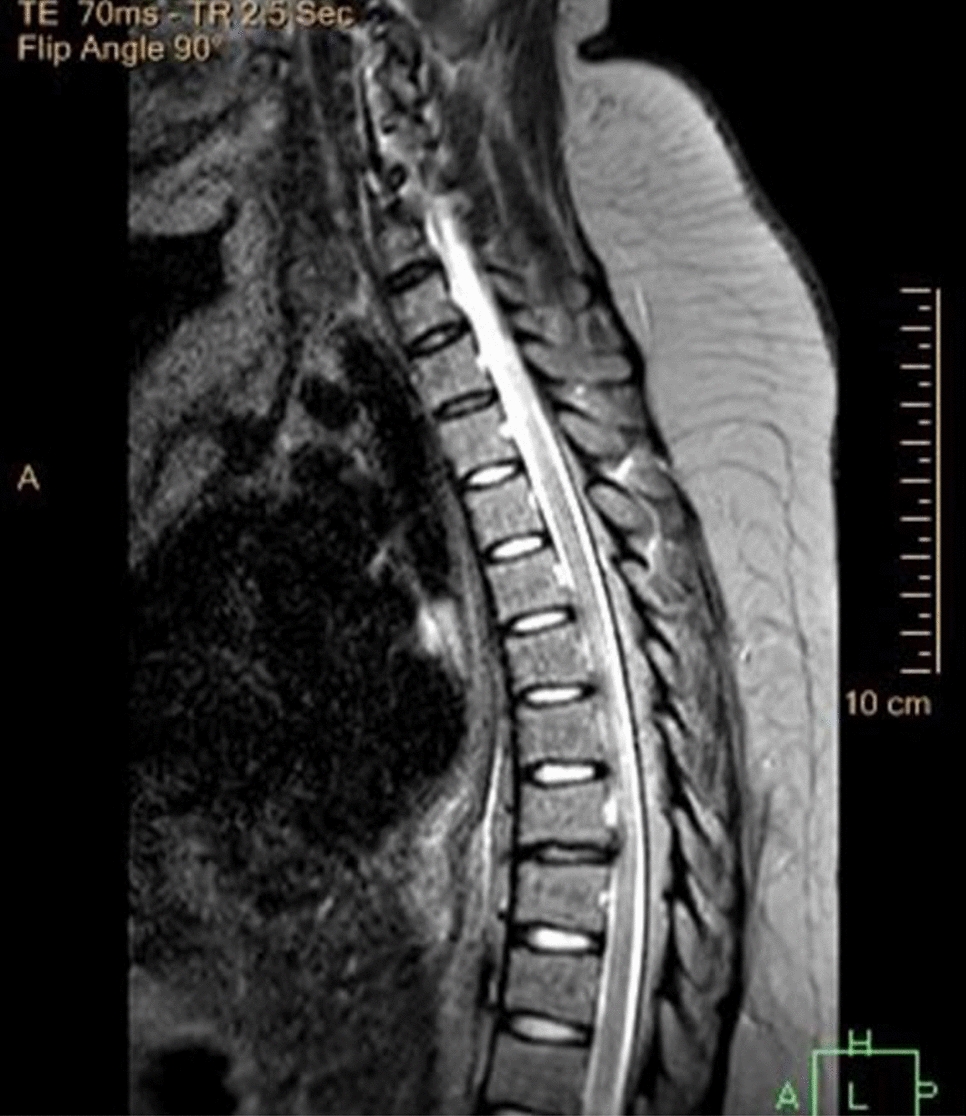
MRI spine technique: T2W1, T1W1 (pre- and post-contrast) sagittal, axial view thoracic area. The apparent loss of cord volume, more prominent at T5 to T7. Associated bilateral paramedian focal, linear T2W/STIR hyperintensity dorsal cord from the in these regions

**Fig. 2:**
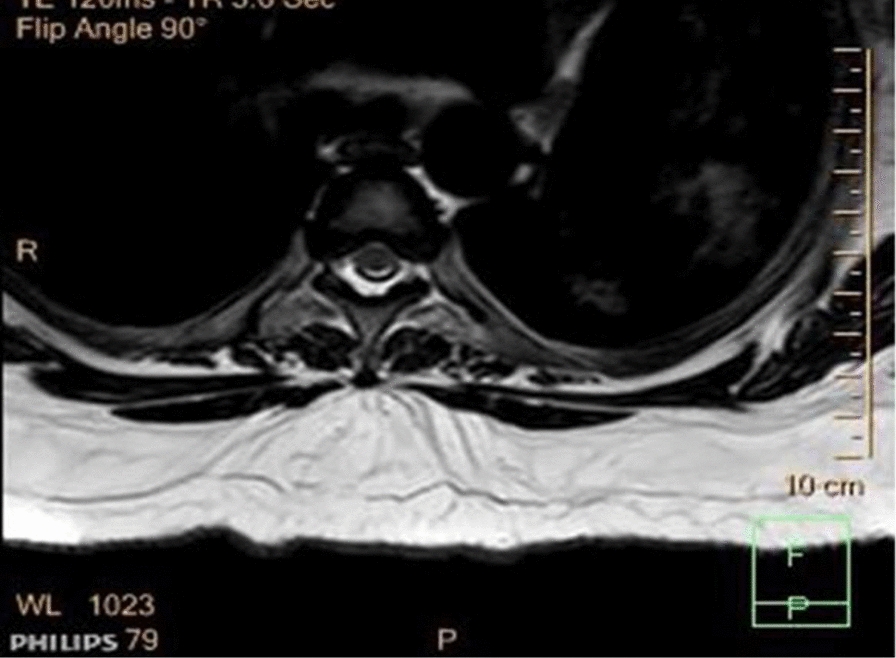
MRI of the spine. Technique T2W1, T1W1 (post-contrast), transverse view shows: STIR intensity in the intramedullary segment of the thoracic spinal cord and loss of volume of the spine more of the posterolateral aspect

The MRI brain was completely normal. The MRI spine however showed diffuse cord atrophy with dorsal signal abnormality involving the lower cervical and thoracic spine, as shown in Figs. 1 and 2. These findings made us think of HIV-VM and subacute combined degeneration of the spinal cord.

We did an extensive serological and CSF workup to exclude both infectious and non-infectious causes of myelopathy. Blood levels for Vitamin B12 and folate were normal (491 pmol/L and 32.7 nmol/L, respectively), her CD4 was 1051 cells/uL with a viral load of < 20copies/ml. Full blood count showed leucocytosis of 17 × 109/L with mild anemia (Hb 11.6 g/dl) and reactive thrombocytosis (platelet: 506 × 109/L) high C reactive protein of 241 mg/L with negative blood cultures. Her U/E revealed hypokalaemia of 2.1 mmol/L and calcium, magnesium, and phosphate were normal. Creatine kinase was normal (20U/L), and her thyroid function tests: TSH: 5.9mIU/L, FT4 12.8 pmol/L.

Her CSF demonstrated high protein: 0.69 g/L, glucose 3.5 mmol/L, polymorphs: 0 cells/uL, lymphocytes: 4 cells/uL, erythrocytes: 240cells/UL.

Repeat CSF showed a negative polymerase chain reaction for viral studies, including herpes simplex virus (HSV), cytomegalovirus (CMV), Epstein–Barr virus (EBV), varicella-zoster virus (VZV), and JC virus. Serum and CSF cryptococcal antigen testing and Bartonella serology were negative. We did not detect CSF antibodies to aquaporin-4 and oligoclonal immunoglobulin bands. CSF VDRL was non-reactive.

We excluded metabolic alkalosis and hypokalaemia, a state of mineralocorticoid excess because of hypertension, and we did a renin/aldosterone ratio, the ratio was 1.28(< 40), CT scan of the adrenal glands was normal.

Considering that there is no clear obstructive, vascular, or neoplastic lesion noted on imaging, the likelihood of the pathology being secondary to direct infection by HIV remains high.

This patient’s condition was further complicated after developing nosocomial pneumonia with type 1 respiratory failure. A Gram-negative bacillus was cultured in her repeat blood and CSF.

Her treatment included Polygam 40 g daily intravenously for 5 days, combined antiretrovirals (cART): abacavir/lamivudine/efavirenz 1 tablet daily orally, piptaz 4,5 g intravenously six-hourly, Aldactone 100 mg orally bd, slow k 2 tablets po twice daily, thiamine 100 mg po daily, pyridoxine 25 mg po daily, and oxygen by facemask. The supportive management of the patient was provided by the physiotherapists and occupational therapists who worked hard. Her case was discussed with the intensive care unit considering her respiratory failure, but she was declared poor prognosis and not accepted.

Her potassium improved to normal, but she remained with power 0/5 in all limbs. Despite IV Immunoglobulin therapy at the higher dosages, the patient did not improve and demised on June 8, 2020.

## Discussion and conclusion

In HIV-VM, there is spinal cord atrophy, which mainly involves the thoracic cord. Sometimes the cervical cord is involved as well [11], in which case patients will present with quadriparesis as our patient did. The lumbar area can be affected in more rare instances. Also, increased T2-weighted signaling of the posterolateral cord usually.

The spinal cord atrophy results from vacuole formation mainly in the posterolateral columns: thereby mostly involving the corticospinal tract and the fasciculus gracilis and cuneatus. (Like vitamin B12 deficiency leading to subacute combined degeneration of the spinal cord. In which case, B12 level in serum is typically low, and those patients respond very well to parenteral B12.)

The exact pathophysiology resulting in the formation of vacuoles (still unclear), but proposed mechanisms include:

- (1) Activation of macrophages in the CNS, which can cause the release of myelotoxic substances or impair the metabolism of Vit B12.
- (2) Oligodendrocyte/myelin injury from the presence of TNF alpha and other cytokines. These will also augment macrophage activity, which can damage myelin.
- (3) Direct infection of astrocytes [13].

Investigations made by Petito et al. confirmed that 26.8% of patients who had autopsy-proven VM had signs and symptoms of VM [14].

The usual HIV-VM presentation is progressive spastic paraparesis, ataxic gait, sphincter disturbance, and erectile dysfunction with no sensory level. Usually occurring in the setting of advanced HIV and sometimes associated with HIV-NCD.

HIV-VM has been documented in the past to be associated with HIV-neurocognitive disorders (HIV-NCD) [12], and sometimes we can see the same damage of the spinal cord in the cerebral hemispheres. In our case, the MRI of the brain was completely normal.

HIV-VM is a diagnosis of exclusion. Therefore, to rule out other causes of myelopathy, an extensive workup must be done.

It is essential to rule out other causes of such a presentation. CSF tests should include cytology, protein level, MCS, CMV, EBV, HSV, TB, JCV, Picornavirus, flavivirus, rhabdovirus, Treponema pallidum, Borrelia, and HIV viral load. MRI is indicated to rule out any other cause of intramedullary/extradural lesions.

CMV polyradiculomyelitis can present like HIV-VM and is usually distinguishable by the absence of spasticity on exam and MRI findings of diffuse, multisegmented signal abnormalities seen in both grey and white matter, with thickening/enhancement of nerve roots [15].

Somatosensory evoked potential (SSEP) may also be supportive if it shows a functional lesion of the spinal cord, but it does not have any pathognomonic pattern [11].

There was no reliable way to confirm vacuolar myelopathy pre-mortem, and the final diagnosis remained a diagnosis of exclusion.

In our center, we currently do not have access to EMG/NCS.

A clinical criterion for diagnosing HIV-VM was created by Chong et al. [11], as shown in Table 1 (Appendix B).

**Table 1:** Table showing the typical case of HIV-VM vs. our case

	Typical presentation	Our patient	
Onset and presentation	Signs and symptoms develop over the subacute–chronic period	Signs and symptoms developed acutely	Atypical
Clinical manifestations			
Motor exam			
Tone	Spastic	Flaccid	Atypical
Power/weakness pattern	Paraparesis, unless the cervical cord is involved, in which case the patient would have quadriparesis	Quadriparesis due to the presence of cervical and thoracic cord atrophy	
Reflexes	Increased	Global areflexia	Atypical
Sensory exam	Usually, no sensory level	Difficult to ascertain due to her confused state, but she did respond to pain and light touch	
GAIT	Ataxic gait	Inability to walk	Atypical
Comorbid status/conditions	In the setting of advanced HIV, however also in cases with normal immune status	Pt was virologically suppressed HIV-VL(< 20 copies/ml), CD5 – 1051 cells/ul	Atypical
	HIV-NCD	Confused but could also be explained by electrolyte imbalance and secondary sepsis. Not simply by HIV-NCD	
Radiological findings	MRI scans are useful in diagnosis; T2-weighted images often show symmetric non-enhancing high signal areas present on multiple contiguous slices, which result from extensive vacuolation (hence the name). Lesions may be confined to the posterior column, especially the gracile tracts, or be diffuse [3, 7]	The MRI spine, however, showed diffuse cord atrophy with dorsal signal abnormality involving the lower cervical and thoracic spine, as shown in Figs. 1 and 2 (Appendix B)	
Treatment options	Symptomatic therapies: cART IVIG [3, 10]	Symptomatic therapies: correction of renal failure, electrolyte imbalance, and treatment of sepsis cART IVIG showed no improvement	Atypical

Our patient presentation was atypical because she was virologically suppressed (VL < 20copies/ml), and her CD4 was 1051 cells/uL. It is important to remember that the CSF viral load measurement would have been essential to check for 'CSF viral escape'. We did not check for HIV-VL in the CSF looking for 'viral escape'. We did not have a large enough CSF sample to send for this test. Her confusion could have been due to HIV-NCD, but since she had nosocomial sepsis and hypokalaemia, it was not the only cause. Also, she had flaccid quadriplegia and absent global reflexes, which is not typical for HIV-VM. Blood showed hypokalaemia, which can explain an associated peripheral neuropathy worsened by HIV infection and subclinical hypothyroidism, but the weakness persisted despite the potassium correction. Vitamin B12 and folate levels were normal; this ruled out a subacute combined spinal cord degeneration. We did an extensive blood and CSF workup for other causes of weakness, which all came back negative. The spinal cord MRI supported the clinical suspicion and showed cord atrophy and T2-weighted hyperintensity of the cervicothoracic spine. We continued her cART and gave her a trial of IVIg, which did not improve the condition. In the ward, she developed nosocomial sepsis and died (Table 1).

The treatment of VM remains an unresolved matter. Therefore, there has been no effective treatment of HIV-VM yet, other than antiretroviral medications. Perhaps, prescribing cART such as zidovudine/abacavir, which has an excellent CNS penetration, should be implemented earlier on in patients with HIV-VM. Another option if the patient is poorly responsive would be to send for CSF-VL and to test for mutations in the CSF. Treatment with L-methionine and IVIg so far has not yielded any beneficial results [16, 17]. Cikurel et al. studied a series of patients with VM to evaluate IVIg's efficacy. They demonstrated that all patients reported reduced palsy in the lower limbs due to the anti-inflammatory effect of IVIg, causing suppression of the complement cascade, inhibition of production of pro-inflammatory cytokines mainly by monocytes, and reinforcing the anti-idiotypic response, leading to the neutralization of growth factors (B-cell), and inhibition of the T-cell proliferation with clonal expansion and activation of T-reg cells and downregulation of the Th17 [10]. Lindst et al. demonstrated that IVIg administration in high doses (30 g/day for 5 days) reduced the latent pool of HIV in the dormant memory CD4 + T-cells [17].

Recently, some authors reported that binding of anti-idiotypic antibodies with epitopes (IgG, and IgM) on the B-lymphocytes and thus inhibiting the production of autoimmune antibodies appears to be the most effective action of IVIg increasing the intrathecal production of oligoclonal Ig, which is a marker for the chronic autoimmune inflammatory process in the CNS [3]. The same author highlighted IVIg treatment's viability in cases presenting HIV-VM, mainly when autoimmune reactions are under suspicion.

We would like to highlight the study done by Cikurel et al. because it is the only report delivering several HIV-VM cases and an actual number of improvements (*n* = 17) after being treated with Ig at the dosage of 2 g/kg for 2 days of IV infusion. Still, they did not report any improvement of spastic paraparesis and urinary incontinence [10], and they did not write the method to measure the improved symptoms and signs. Other authors also declare a complete recovery of HIV-VM symptoms and signs after being treated with highly active antiretroviral therapy [18, 19]. Still, the procedure to measure the improvements is also questionable.

Reviewed in this manuscript is an atypical HIV-VM presentation (she presented with flaccid quadriparesis, which was acute, and had well-controlled HIV) and did not respond to intravenous immunoglobulin (IVIg). Even though our patient did not show any signs of improvement after IVIg therapy, to perform a placebo-controlled clinical trial of IVIg in patients with HIV-VM can be other recommendations to confirm or deny the real benefits of this medication.

## Data Availability

Data used in this study are available on reasonable request from the corresponding author.

## Abbreviations

ABC/3TC/EFV: Abacavir/ lamivudine/efavirenz
BMI: Basal metabolic index
cART: Combined antiretroviral therapy
CMV: Cytomegalovirus
CSF: Cerebro-spinal fluid
CSF-VL: Cerebro-spinal fluid viral load
CT: Computed tomography
EBV: Epstein–Barr virus
EMG: Electromyography
FT4: Thyroxine
HAART: Highly active antiretroviral therapy
Hb: Hemoglobin
HIV: Human immunodeficiency virus
HIV-NCD: Human immunodeficiency virus-associated neurocognitive disorders
HIV-VL: Human immunodeficiency virus-associated viral load
HIV-VM: Human immunodeficiency virus-associated vacuolar myelopathy
HTLV: Human T-cell lymphotropic virus type 1
HSV: Herpes simplex virus
IVIg: Intravenous immunoglobulins
JCV: John Cunningham virus
MCS: Microscopy, culture, sensitivity
MRI: Magnetic resonance imaging
NCS: Nerve conduction studies
NMACH: Nelson Mandela Academic Central Hospital
TSH: Thyroid stimulating hormone
U/E: Urea and electrolytes
VDRL: Venereal disease research lab test
VL: Viral load
VZV: Varicella-Zoster virus
